# Supplementary material for: Efficient Purification of R-phycoerythrin from Marine Algae (Porphyra yezoensis) Based on a Deep Eutectic Solvents Aqueous Two-Phase System
Source: Mar Drugs. 2020 Dec 4;18(12):618. doi: 10.3390/md18120618 (PMC7761831; doi:10.3390/md18120618)
Supplement: Supplementary file 1 [file marinedrugs-18-00618-s001.pdf]

Supplementary Material

A Green Purification Method of R-phycoerythrin from Marine Algae (*Porphyra. yezoensis*) based on Deep Eutectic Solvents Aqueous Two-Phase System

Yifeng Xu[1], Quanfu Wang[1,2,*], Yanhua Hou[2,*]

[1]*School of Environment, Harbin Institute of Technology, Harbin 150090, P.R. China*

[2]*School of Marine Science and Technology, Harbin Institute of Technology, Weihai 264209, P.R. China*

*Corresponding authors: Quanfu Wang; Yanhua Hou

E-mail addresses: wangquanfuhit@hit.edu.cn (Quanfu Wang);

houyanhuahit@hit.edu.cn (Yanhua Hou)

Telephone number: +86-631-6587240.

Fax: +86-631-6587240.

Contents

Figure S1. Infrared spectroscopy of six deep eutectic solvents: (a) choline chloride-ethylene glycol (ChCl-EG), (b) choline chloride-D-sorbitol (D-ChCl-S), (c) choline chloride-glycerol (ChCl-G), (d) choline chloride-D-glucose (D-ChCl-Gl), (e) choline chloride-D-fructose (D-ChCl-F), (f) choline chloride-urea (ChCl-U). 3

Table S1. The results of the precision, repeatability and stability experiments...................... 4

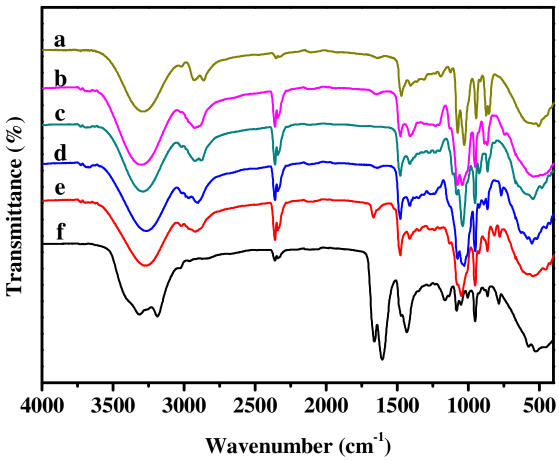

Figure S1. Infrared spectroscopy of six deep eutectic solvents: (a) choline chloride-ethylene glycol (ChCl-EG), (b) choline chloride-D-sorbitol (D-ChCl-S), (c) choline chloride-glycerol (ChCl-G), (d) choline chloride-D-glucose (D-ChCl-Gl), (e) choline chloride-D-fructose (D-ChCl-F), (f) choline chloride-urea (ChCl-U).

Table S1. The results of the precision, repeatability and stability experiments.

**Precision experiment results (n = 5)**					
Repeats	1	2	3	4	5
Absorbance (565 nm)	0.630	0.571	0.598	0.588	0.544
E (%)	93.92	92.10	94.07	93.17	94.13
RSD (%)			0.92		
**Repeatability experiment results (n = 5)**					
Sample number	1	2	3	4	5
Absorbance (565 nm)	0.551	0.540	0.537	0.532	0.538
E (%)	93.65	92.47	93.31	91.97	93.80
RSD (%)			0.85		
**Stability experiment results (n = 5)**					
Day number	1	2	3	4	5
Absorbance (565 nm)	0.612	0.647	0.631	0.652	0.650
E (%)	92.89	92.51	91.87	91.25	91.57
RSD (%)			0.73		

[1] $E=(C_tV_t)/(C_tV_t+C_bV_b)$, where C_t and C_b are the concentrations of the R-PE in the DES-rich top phase and salt-rich bottom phase. V_t and V_b represent the volume of the top phase and the bottom phase.

2. RSD represents relative standard deviation.
